# Supplementary material for: Severe COVID-19 patients have impaired plasmacytoid dendritic cell-mediated control of SARS-CoV-2
Source: Nat Commun. 2023 Feb 8;14:694. doi: 10.1038/s41467-023-36140-9 (PMC9907212; doi:10.1038/s41467-023-36140-9)
Supplement: Supplementary file 4 — Source Data [file 41467_2023_36140_MOESM4_ESM.zip › Source data/Venet Fig 2b_mDC1s_def/2022-04-28_Thu_17-00-00_GradientBoostClassifier_9/Confusion_matrix_Downsampling 9.pdf]

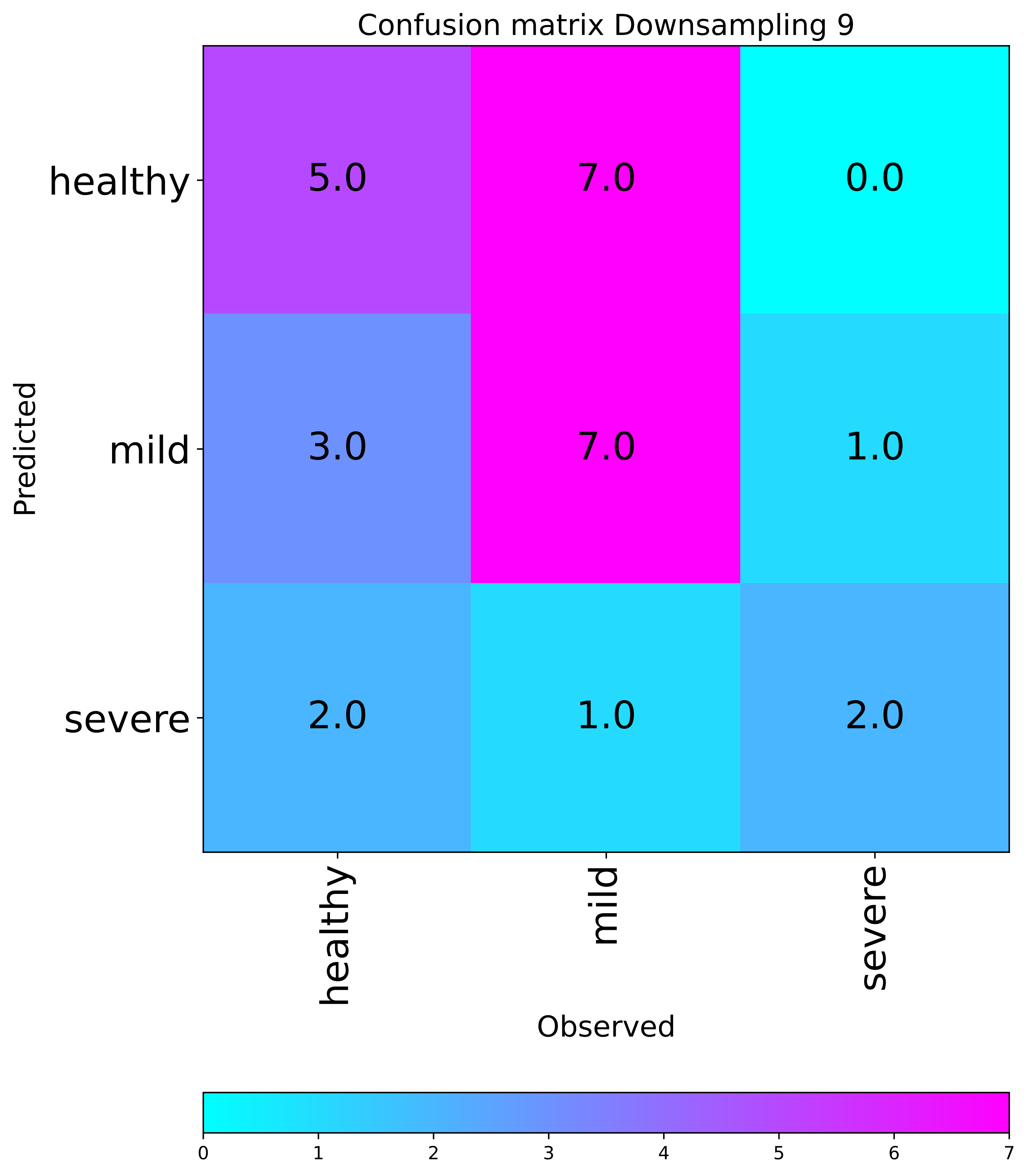
Confusion matrix Downsampling 9
healthy
mild
severe
Predicted
5.0
7.0
0.0
3.0
7.0
1.0
2.0
1.0
2.0
healthy
mild
severe
Observed
0
1
2
3
4
5
6
7
